# Photoelectric detection of electron spin resonance of nitrogen-vacancy centres in diamond

**DOI:** 10.1038/ncomms9577

**Published:** 2015-10-21

**Authors:** E. Bourgeois, A. Jarmola, P. Siyushev, M. Gulka, J. Hruby, F. Jelezko, D. Budker, M. Nesladek

**Affiliations:** 1 IMOMEC division, IMEC, Wetenschapspark 1, B-3590 Diepenbeek, Belgium; 2 Institute for Materials Research (IMO), Hasselt University, Wetenschapspark 1, B-3590 Diepenbeek, Belgium; 3 Department of Physics, University of California, Berkeley, California 94720-7300, USA; 4 Institute for Quantum Optics and IQST, Ulm University, Albert-Einstein-Allee 11, D-89081 Ulm, Germany; 5 Czech Technical University in Prague, Sitna sq. 3105, 272 01, Kladno, Czech Republic; 6 Helmholtz Institute, Johannes Gutenberg University, D-55099, Mainz, Germany

## Abstract

The readout of negatively charged nitrogen-vacancy centre electron spins is essential for applications in quantum computation, metrology and sensing. Conventional readout protocols are based on the detection of photons emitted from nitrogen-vacancy centres, a process limited by the efficiency of photon collection. We report on an alternative principle for detecting the magnetic resonance of nitrogen-vacancy centres, allowing the direct photoelectric readout of nitrogen-vacancy centres spin state in an all-diamond device. The photocurrent detection of magnetic resonance scheme is based on the detection of charge carriers promoted to the conduction band of diamond by two-photon ionization of nitrogen-vacancy centres. The optical and photoelectric detection of magnetic resonance are compared, by performing both types of measurements simultaneously. The minima detected in the measured photocurrent at resonant microwave frequencies are attributed to the spin-dependent ionization dynamics of nitrogen-vacancy, originating from spin-selective non-radiative transitions to the metastable singlet state.

## Abstract (web summary)

Nitrogen-vacancy colour centre defects in diamond are one possible host for qubits, but such an application requires a method for reading out the colour centre spin state. Here, the authors demonstrate a photoelectric readout technique of the magnetic resonances of these colour centres.

## Graphical abstract

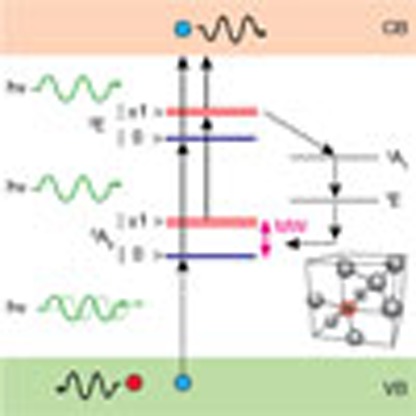

Reading out the electron spin of the negatively charged nitrogen-vacancy (NV^−^) centres in diamond is essential for applications in quantum computing and secure communication^1^,^2^, as well as for nanoscale magnetic and electric sensing^3^,^4^,^5^ and for non-perturbing sensing and imaging of quantum objects^6^,^7^. NV^−^ ensembles in bulk diamond are in addition used for high-sensitivity magnetometry, with submicrometre resolution^8^. At present, the readout of NV^−^ centres electron spin state is typically performed optically, by detecting photons using confocal microscopy. No all-diamond readout technique allowing for integration with diamond electronic chips is available. One of the limiting factors for optical detection is the low collection efficiency of photons emitted by NV^−^ centres in bulk diamond, due to the limitations of objective optics and to the high index of refraction of diamond. Reaching an optical detection efficiency higher than a few percents most often requires complex microfabrication^9^,^10^, even though simpler techniques for the fabrication of diamond photonics structures were recently proposed^11^. Additionally, the use of NV^−^ centres for quantum computing at room temperature is based on the entanglement between the spins of adjacent NV^−^ centres at a distance of the order of 10 nm^12^. The individual optical readout of such proximal NV^−^ centres requires a resolution below the diffraction limit, which can only be achieved by complex optical techniques such as stimulated emission depletion (STED) microscopy^13^ or reversible saturable optical linear fluorescence transitions (spin-RESOLFT) microscopy^14^.

In the following, we describe an alternative non-optical technique consisting in the direct photoelectric readout of the electron-spin resonance of NV^−^ centres. This scheme is denoted as photocurrent detection of magnetic resonance (PDMR). Similarly to the readout method presented in ref. 15, our detection technique is based on the spin-dependence of NV^−^ ionization dynamics. However, while in ref. 15 the spin detection relies on an optical readout of NV charge state, our method is based on direct electric detection of charge carriers excited from NV^−^ centres to the diamond conduction band, which can be performed electrically on a chip. In contrast to a recently proposed scheme, in which electronic readout of NV^−^ centres spin state is performed by monitoring non-radiative energy transfers to graphene^16^, PDMR detection can be performed in an all-diamond device, without the indirect energy transfer to another material. Photoelectric detection of spin resonances only requires the fabrication of electrodes on the diamond chip by standard lithography. It avoids the complexity of confocal imaging and would allow for example the detection of NV^−^ spin resonance in light-scattering media. We demonstrate the PDMR technique on NV^−^ ensembles; however, it may be developed to be used for the readout of single NV^−^ centres spins. By further downscaling the inter-electrode distance to 10 nm gaps^17^,^18^, PDMR has the potential to reach the independent readout of NV^−^ centres situated closer than the diffraction limit. Another peculiar feature of the photocurrent detection principle is the photoelectric gain mechanism^19^,^20^, which might lead to high detection efficiency since every photon has the ability to generate more than one electron–hole pair.

## Results

### Demonstration of PDMR on type-Ib diamond

**Figure 1:**
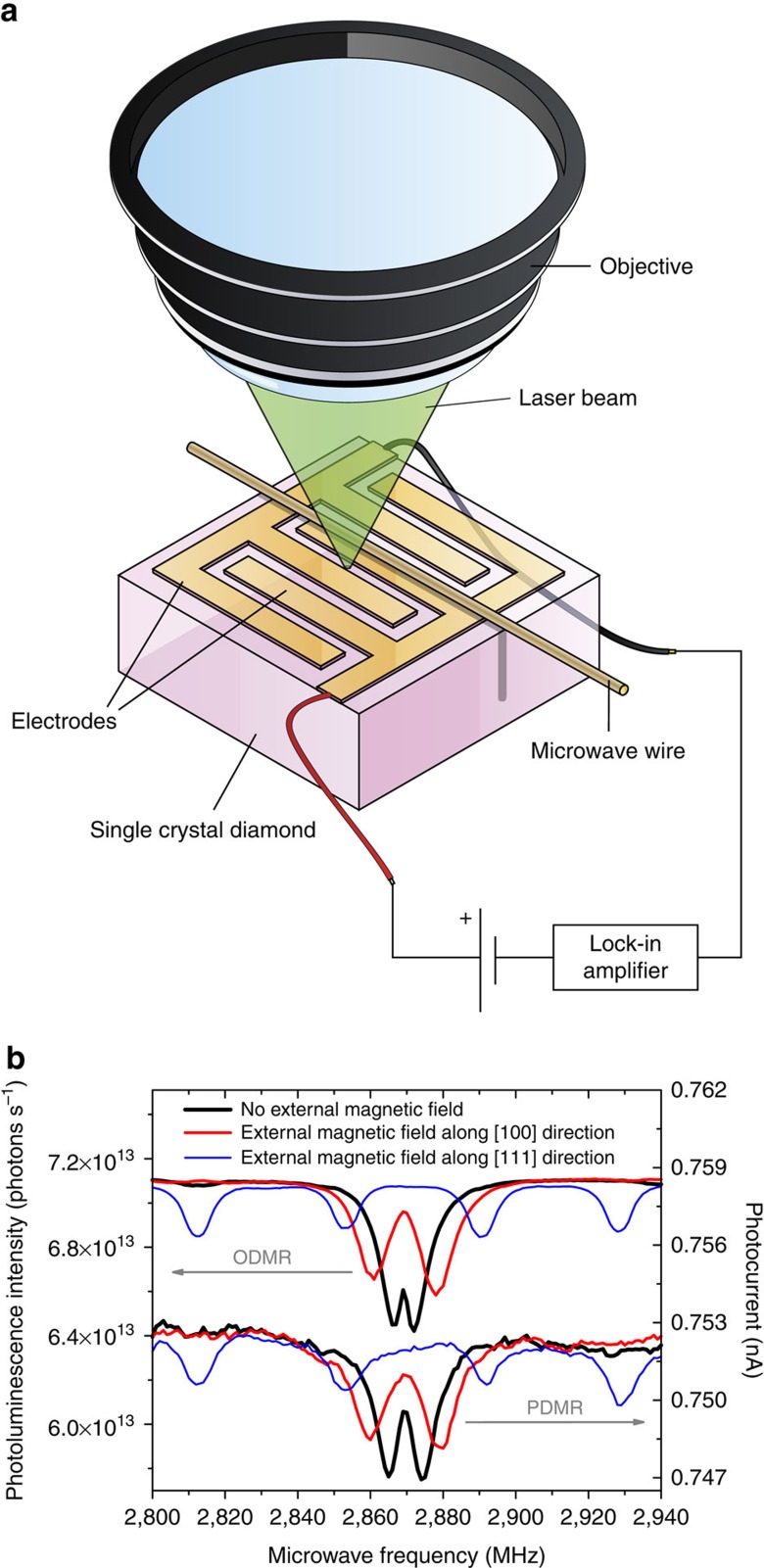
PDMR on NV centres in type-Ib diamond. (**a**) Schematic representation of the setup used for PDMR. (**b**) Comparison of ODMR and PDMR spectra recorded simultaneously. Sample E2, green light power: 100 mW, applied electric field: 5 × 10^4^ V cm^−1^, light pulse duration: 940 μs, distance between contacts: 100 μm, magnitude of the external magnetic field: 0.50 mT along the [100] direction, 2.0 mT along the [111] direction.

A schematic representation of the PDMR setup is depicted in Fig. 1a. The principle of PDMR is demonstrated on NV^−^ centres ensembles, by characterizing an irradiated and annealed type-Ib single-crystal diamond plate of [100] crystallographic orientation (sample E2), containing both NV^−^ centres (∼20 p.p.m.) and substitutional nitrogen centres (N_s_^0^, ∼200 p.p.m.). Optical detection of magnetic resonance (ODMR) and PDMR were performed simultaneously on this sample. For this, the intensity of photoluminescence and the photocurrent were measured simultaneously while scanning the microwave frequency in the absence and in the presence of an external magnetic field (Fig. 1b). Minima in the photoluminescence intensity are observed at microwave frequencies inducing resonant transitions between the |0> and the |±1> spin sublevels of the NV^−^ spin triplet ground state (^3^A_2_)^21^. Minima in photocurrent are clearly detected at identical frequencies, demonstrating that photocurrent measurements can be used to detect the spin resonances of NV^−^ centres.

The two resonances observed in ODMR and PDMR spectra in the absence of an external magnetic field indicate the existence of a splitting between linear combinations of the |+1> and |−1> spin sublevels of NV^−^ (hereafter referred to as ‘|±1> spin manifold'), induced by local strain in the material^8^ and, in the case of PDMR measurements, by the externally applied electric field. We will refer to this splitting between magnetic resonances at zero-magnetic field as ‘zero-field splitting' (ZFS). The origin of the difference between the ZFS observed in ODMR and PDMR is discussed in the Supplementary Note 1. In the presence of an external static magnetic field applied using a permanent magnet, a further splitting of resonant frequencies is observed both in ODMR and PDMR spectra, reflecting the perturbation of the |±1> spin manifold of NV^−^ ground state because of the Zeeman effect. As expected^22^, two magnetic resonances are observed when the magnetic field is applied along the [100] direction of the diamond crystal and four when it is applied along the [111] direction (Fig. 1b).

### Investigation of PDMR mechanism

**Figure 2:**
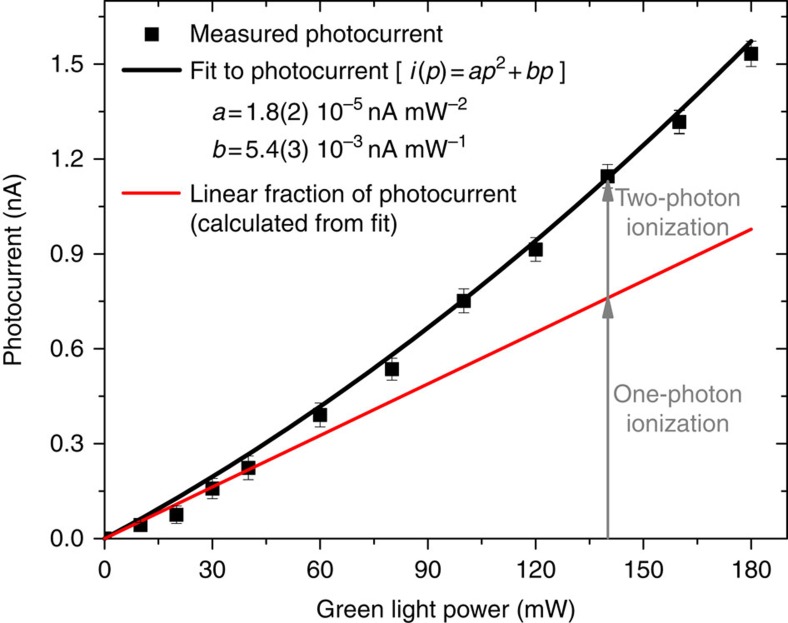
Light power dependence of photocurrent in type-Ib diamond. Sample E2, applied electric field: 5 × 10^4^ V cm^−1^, light pulse duration: 940 μs, distance between contacts: 100 μm. *i*: photocurrent, *p*: optical power, *a* and *b*: fitting prefactors. Error bars represent the s.d. from the mean of 32 photocurrent measurements.

**Figure 3:**
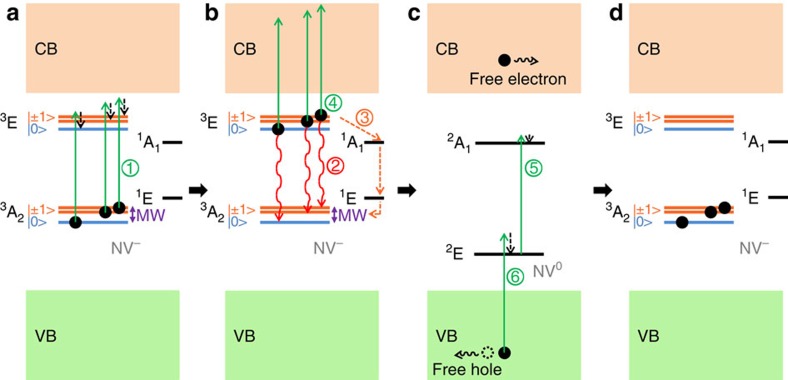
Mechanism proposed to explain PDMR measurements. A simplified electronic energy level scheme (not to scale) of the NV^−^ centre at room temperature is represented (MW: microwave). (**a**) The absorption of a first photon promotes an electron from the ^3^A_2_ ground state to the ^3^E state of NV^−^ (transition (1)). (**b**) From ^3^E, electrons can radiatively decay back to ^3^A_2_ (transition (2)) or be excited to the diamond conduction band (CB) by the absorption of a second photon (transition (4)). The spin-selective non-radiative decay of electrons to the singlet state ^1^A_1_ (transition (3)), followed by transition to the metastable state ^1^E, enables PDMR and ODMR. (**c**) The two-photon ionization of NV^−^ results in the formation of a NV^0^ centre and a free electron in the CB. By a two-photon process in which the NV^0^ centre is first excited (transition (5)) and an electron is then promoted from the valence band (VB) to the vacated orbital of NV^0^ (transition (6)), the NV centre can finally be converted back to its negatively charged state (**d**).

**Figure 4:**
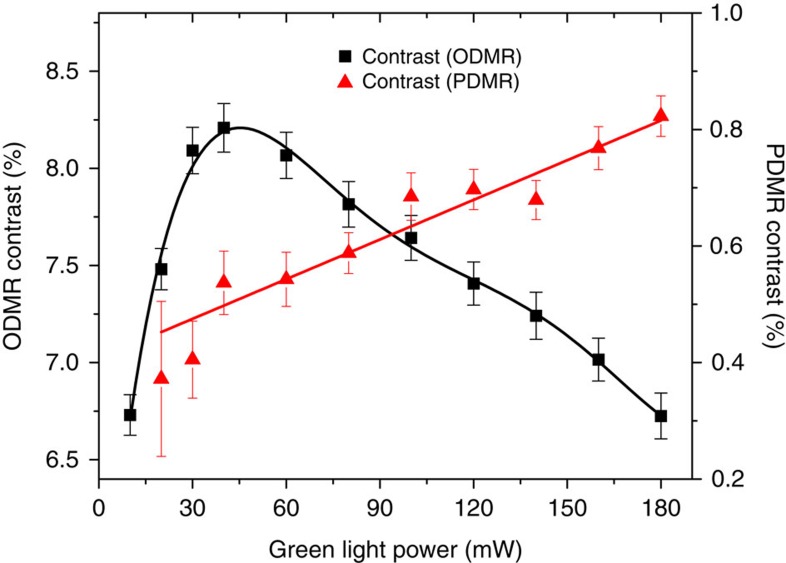
Light power dependence of PDMR and ODMR constrasts. Sample E2, applied electric field: 5 × 10^4^ V cm^−1^, light pulse duration: 940 μs, distance between contacts: 100 μm. Error bars represent the s.e.m. of the fitted values (see Methods for details on the fitting procedure). Solid lines are guides for the eye.

**Figure 5:**
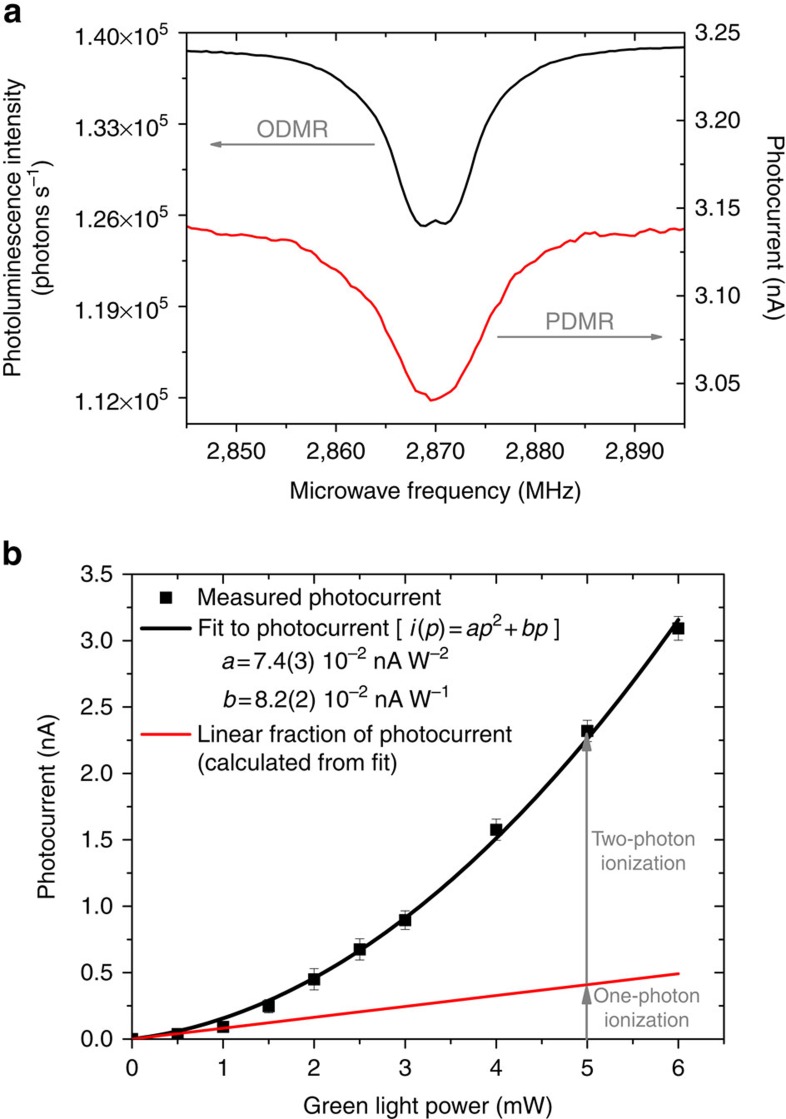
PDMR on NV centres in type-IIa diamond. Sample E7, light pulse duration: 940 μs, applied electric field: 3.3 × 10^4^ V cm^−1^, distance between contacts: 15 μm. (**a**) Comparison of ODMR and PDMR spectra measured simultaneously in the absence of external magnetic field (green light power: 6 mW). (**b**) Photocurrent as a function of the 532 nm light power. *i*: photocurrent, *p*: optical power, *a* and *b*: fitting prefactors. Error bars represent the s.d. from the mean of measured values.

To investigate the photoionization mechanism inducing the photocurrent minima at NV^−^ spin-resonance frequencies, the photocurrent detected on sample E2 was measured as a function of the green light power (Fig. 2). A good fit to the experimental data is obtained using the sum of a linear and a quadratic function, indicating that the measured photocurrent results from the combination of a one-photon and a two-photon ionization processes^23^. In previous experiments^23^ and in theoretical studies^24^,^25^ it was demonstrated that a photon energy higher than 2.6 eV is necessary to induce the photoionization of NV^−^ via a one-photon process, that is, to directly promote an electron from the ground state of NV^−^ to the conduction band. Based on this argument and on the quadratic power dependence of the photocurrent, we conclude that a two-photon absorption process is responsible for the NV^−^-related part of the photocurrent induced by green light (2.33 eV). The mechanism for the two-photon ionization of NV^−^ centres in diamond has been experimentally established^23^,^26^,^27^ and modelled^27^. In this process, the absorption of a first photon promotes an electron from the ^3^A_2_ triplet ground state of NV^−^ to its ^3^E triplet excited state (Fig. 3a, transition (1)) and a second photon excites this electron to the conduction band of diamond (Fig. 3b, (4)), which results in the conversion of the NV centre to its neutral state NV^0^ and in the promotion of an electron into the conduction band (Fig. 3c). To ensure the charge neutrality in the PDMR detection circuit, the NV^0^ centres formed by ionisation of NV^−^ have to be subsequently converted back to the NV^−^ state (Fig. 3d), either by capturing an electron from a donor defect (in particular from N_s_^0^, present in high concentration in the type-Ib sample under study)^28^,^29^ or by two-photon conversion from NV^0^ to NV^−^. In the latter process, the absorption of a first photon excites the NV^0^ centre (Fig. 3c, (5)), while a second photon promotes an electron from the valence band to the vacated orbital of NV^0^ (Fig. 3c, (6)), leaving a hole in the valence band^23^,^27^. This photo-induced process does not require the presence of electron-donor defects in the diamond crystal.

The linear contribution to the measured photocurrent (Fig. 2) is likely associated with one-photon ionization of N_S_^0^. N_S_^0^ is indeed the dominant point defect in the E2 sample, and the threshold energy for the photoionization of this defect^30^,^31^ is well below the 2.33 eV excitation energy used in our experiment.

The mechanism proposed to explain the photoelectric detection of NV^−^ magnetic resonances is presented in Fig. 3. The minima observed in the photocurrent at resonant microwave frequencies inducing transitions from the |0> to the |±1> electron spin sublevels of NV^−^ ground state (^3^A_2_) indicate that the photoionization dynamics of NV^−^ is spin-dependant. In ODMR, electrons are initially pumped into the ^3^A_2_ |0> spin state under the effect of green illumination, from which they are coherently driven to the ^3^A_2_ |±1> spin sublevels by the resonant microwave field^32^. Photoluminescence originates from the radiative decay of electrons from the excited state (^3^E) to the ground state (Fig. 3b, (2)). Because of spin-selective intersystem crossing^33^, electrons in the ^3^E |±1> sublevels can decay non-radiatively to the ^1^A_1_ singlet state (lifetime ≤ 1 ns)^34^ from which they further fall into the ^1^E metastable singlet state (electron shelving). This non-radiative decay path (Fig. 3b, (3)) induces a difference between the brightness of the transitions associated with the different NV^−^ spin sublevels, which provides the contrast monitored by ODMR. For PDMR, we expect that electron shelving is also the dominant mechanism explaining the observed contrast. Electrons excited to the ^3^E |±1> sublevels have a non-zero probability to undergo the shelving transitions via the ^1^A_1_ and ^1^E states. For the time during which an electron initially in the ^3^A_2_ |±1> state undergoes these non-radiative transitions, it does not contribute to the photocurrent. Specifically, the ^1^E metastable state has a lifetime of 220 ns at room temperature^34^. For that period, the ^1^E state stores the electron which leads to a temporary decrease in the occupation of NV^−^ ground state and reduces the rate of two-photon ionization (proportional to the occupation of ^3^A_2_). This process probably induces the observed magnetic resonances in photocurrent. A similar mechanism was proposed recently to explain the spin-dependence of NV^−^ ionization dynamics^15^.

An alternative photoionization path would be the direct promotion of electrons from the ^1^E metastable state to the conduction band. This ionization path would lead to a positive sign of PDMR, since electrons in the ^3^E |±1> sublevels have a higher probability to decay non-radiatively to the singlet state, which leads to a higher occupation of the ^1^E metastable state. However, the negative resonances observed in PDMR spectra (Fig. 1b) suggest that the contribution of this photoionization process to the total photocurrent at the resonant microwave frequency is significantly lower than the contribution of direct transitions from the excited state ^3^E to the conduction band.

### PDMR contrast

Figure 4 depicts the light power dependence of the ODMR and PDMR contrasts measured on sample E2. The maximal observed contrasts are ∼0.8% for PDMR and 8.2 % for ODMR. This is partly due to the fact that only the two-photon fraction of the photocurrent (associated with the ionization of NV^−^) gives rise to detectable electron spin resonances, while its linear fraction is not affected by the microwaves. In ODMR, the contrast is observed to decrease for green light powers above 45 mW, which can be explained by the fact that at strong light power the electron spin polarisation rate becomes larger that the Rabi frequency of the microwave driving field^35^. In the case of PDMR measurements performed on type-Ib diamond, a monotonous increase of the PDMR contrast with the light intensity is observed (Fig. 4). The absence of saturation detected in the PDMR contrast can be explained by the fact that increasing the light power leads to an increase in the quadratic fraction of the photocurrent (associated with ionization of NV^−^ centres) with respect to the linear fraction (associated with ionization of N_S_^0^). However, while the quadratic fraction of the photocurrent increases by a factor four between 30 and 180 mW (Fig. 2), the PDMR contrast only increases by a factor of two, which seems to reflect the fact that as the optical power increases, the spin polarization rate becomes higher than the Rabi frequency, as discussed above. For a quantitative evaluation of this phenomenon, the rate of shelving transitions to the ^1^E singlet state and the two-photon ionization rate have to be further investigated. The comparison between the light power-dependence of the linewidths of ODMR and PDMR resonances is presented in Supplementary Fig. 1 and commented in Supplementary Note 2.

### Influence of the applied electric field

Since the photoelectric detection technique requires the application of a DC electric field to drive the generated charge carriers toward electrodes, the influence of the magnitude of the applied electric field on PDMR spectra was studied. The presence of an external electric field affects the electronic spin sublevels of NV^−^ centres ground state, due to the Stark effect^36^,^37^,^38^ . Normalized ODMR and PDMR spectra recorded under different electric fields on sample E2 are presented in Supplementary Fig. 2. No measurable effect of the external electric field on the ODMR signal is detected, even under high applied electric field (5 × 10^4^ V cm^−1^). Concerning PDMR measurements, it can be observed that in spite of a decrease in the photocurrent, magnetic resonances are clearly detected under an applied electric field as low as 1.3 × 10^2^ V cm^−1^, corresponding to a local electric field of 3.3 × 10^2^ V cm^−1^ in diamond. Considering the axial and transverse electric susceptibility parameters of 0.35 and 17 Hz cm V^−1^, respectively, reported in ref. 36, this local electric field applied along the axis of an NV^−^ centre leads to a splitting at zero-magnetic field of 5.7 kHz between the two proper eigenstates in the |±1> manifold and to a shift of 120 Hz of the splitting between the |0> and the |±1> spin sublevels^38^, which is orders of magnitude lower than the full-width at half-maximum (FWHM) of the detected resonances. The impact of the applied electric field on PDMR linewidth and ZFS is presented in Supplementary Fig. 3a,b, and discussed in Supplementary Note 1.

### PDMR on type-IIa diamond with low NV^−^ concentration

To demonstrate the possibility of performing PDMR with NV^−^ ensembles in type-IIa diamond with low N_S_^0^ concentration, we have used an electron-irradiated and annealed optical grade diamond with estimated N_S_^0^ concentration below 1 p.p.m. and NV^−^ concentration around 10 p.p.b. (sample E7). We were able to detect the NV^−^ spin electron resonance in the photocurrent measured on this sample (PDMR spectrum compared with ODMR spectrum in Fig. 5a). The light power-dependence of the photocurrent detected on this sample is presented in Fig. 5b. The experimental data can be fitted with the sum of a quadratic function (corresponding to a two-photon ionization process) and a linear function (corresponding to a one-photon ionization process). By comparing with the photocurrent measured on type-Ib diamond (Fig. 2), it appears that the ratio between the linear and quadratic prefactors *b* and *a* (see Fig. 5b) is about 300 times lower for type-IIa diamond than for type-Ib diamond. This suggests that in the volume of sample E7 contributing to the photocurrent, the concentration of N_s_^0^ defects is low and that a nearly pure two-photon ionization process occurs, without major contribution from the ionization of N_S_^0^ defects to the photocurrent. Correspondingly, a higher PDMR contrast is obtained on type-IIa than on type-Ib diamond. Indeed, a PDMR contrast of 3% was observed on type-IIa diamond in optimal conditions (corresponding to 3 % of the laser power and 0.8 % of the microwave power leading to optimal contrast on type-Ib diamond). This result shows that, as expected, the PDMR technique is more efficient on type-IIa diamond than on type-Ib diamond, due to the lower contribution of N_S_^0^ photoionization to the total photocurrent.

### PDMR sensitivity

To assess the sensitivity of PDMR detection and its potential use for the characterization of single NV^−^ centres, the efficiency *η* of charge carrier creation was estimated. In a first approximation, the magnitude of the electric field is considered as uniform in the diamond crystal and the field lines are considered as half ellipses extending between electrodes. The photocurrent associated with the two-photon ionization of NV^−^ centres (quadratic fraction of the photocurrent) *i*_NV_ can be expressed as


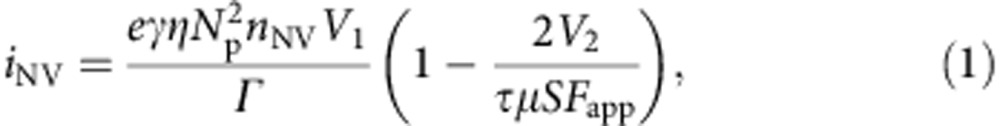


where *e* is the elementary charge (C), *γ* the photoelectric gain, *N*_p_ is the number of incident photons per second (s^−1^), *n*_NV_ the density of NV^−^ centres in the sample (cm^−3^), *V*_1_ the volume containing the NV^−^ centres contributing to the photocurrent (cm^3^), *Γ* the decay rate from NV^−^ excited state to NV^−^ ground state (s^−1^), *V*_2_ the volume in which the drift of free charge carriers takes place (cm^3^), *τ* the recombination lifetime of charge carriers (s), *μ* the electron mobility (cm^2^ V^−1^ s^−1^), *S* the axial cross section of the generation volume *V*_1_ (cm^2^) and *F*_app_ is the applied electric field (V cm^−1^). The derivation of this expression and the evaluation of the different parameters contained in the formula are detailed in the Supplementary Note 3.

It should be noted that contrary to photoluminescence, the photoelectron generation will not saturate at high pumping power, since the ^3^A_2_ ↔ ^3^E transition and the subsequent transition from ^3^E to the conduction band do not saturate. The photocurrent is therefore only limited by the reformation of NV^−^ centres from NV^0^ centres by re-pumping electrons from the valence band.

The gain *γ* of a photoconductor detector with ohmic contacts is defined as the number of charges collected at electrodes for each photogenerated charge carrier^19^. If the lifetime of one of the charge carriers is longer than its transit time, it will be able to transit several times in between electrodes before recombining. During this time, the boundary conditions (continuity of the current) for ohmic electrodes force one of the electrodes to provide charge carriers of the opposite polarity. In case of charge carriers induced by the photoionization of NV^−^ centres, the process continues as long as the hole remains localized on the NV centre. The photoelectric gain *γ* is therefore equal to the ratio between the recombination lifetime *τ* and the electron transit time *t*_transit_^19^. The transit time *t*_transit_ (s) can be expressed as a function of the electron mobility *μ* (cm^2^ V^−1^ s^−1^), the distance *L* between electrodes (cm) and the voltage *U* applied between electrodes (V):


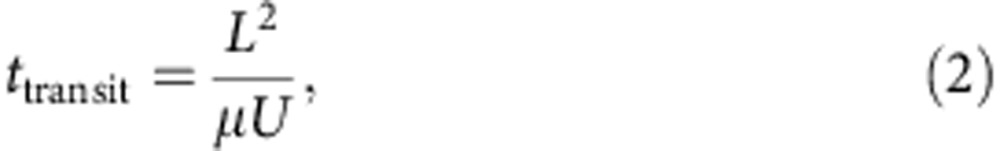


which leads to the following expression for the photoelectric gain:


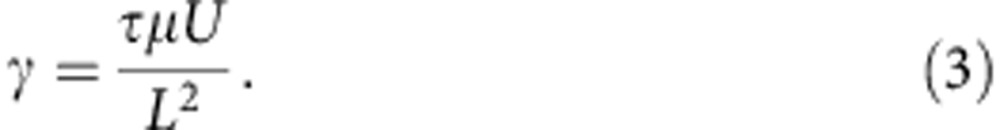


For the type-Ib diamond (sample E2), we assume a charge carrier mobility^39^ of 3.6 × 10^2^ cm^2^ V^−1^ s^−1^ and a lifetime between 80 ps and 3 ns^40^. Based on the electron mobility and lifetime reported for ultra-high purity type-IIa diamond^41^, we assume a charge carrier mobility between 4.5 × 10^2^ and 4.5 × 10^3^ cm^2^ V^−1^ s^−1^ and a charge carrier lifetime between 100 ns and 2 μs for the irradiated and annealed optical grade type-IIa diamond under study (sample E7). Considering these ranges of mobility and lifetime, we estimate a photoelectric gain between 1 and 40 for measurements performed on type-Ib diamond and between 1 × 10^3^ and 2 × 10^5^ for type-IIa diamond. The higher photocurrent detected per NV^−^ centre for type-IIa diamond (1 × 10^−15^ A per NV^−^ centre under 6 mW illumination) than for type-Ib diamond (2 × 10^−19^ A per NV^−^ centre under 100 mW illumination) results therefore partly from the higher photoelectric gain.

Equation (1) has been used to estimate the efficiency of charge generation *η*, based on the quadratic fraction of the photocurrent measured with contacts separated by 15 μm and considering the ranges of charge mobility and charge carrier lifetime mentioned above. In the case of a single NV^−^ centre implanted in electronic grade CVD diamond, a very high photoelectric gain can theoretically be obtained, due to the higher lifetime (up to 2 μs) and mobility (up to 4500, cm^2^ V^−1^ s^−1^) of charge carriers^41^. Assuming the mobility and lifetime mentioned above, a distance of 10 nm between electrodes^17^,^18^ and an applied electric field of 5 × 10^4^ V cm^−1^, a gain of 5 × 10^8^ can theoretically be expected. Considering the efficiency of charge creation η determined on the type-IIa diamond under study and a photoelectric gain of 5 × 10^8^, the two-photon ionization of a single NV^−^ centre illuminated at *I*_sat_ (light intensity necessary to saturate the ^1^E singlet state, estimated^42^ to be ∼600 MW m^−2^) would induce a photocurrent between 6 fA and 1 pA, which is measurable, for example by lock-in amplification. Our calculation indicates therefore that PDMR could potentially be used for the characterization of single NV^−^ centres in electronic grade type-IIa diamond.

### PDMR bandwidth

To investigate the bandwidth of the photoelectric detection technique and to demonstraste the possibility to employ it for pulsed spin detection experiments (Ramsey or Rabi oscillations, spin-echo experiments and so on), faster photocurrent measurements were performed on the E2 sample (see Supplementary Note 4). The photoelectric detection of spin magnetic resonances was achieved using light pulses down to 10 μs, with lock-in amplification of the photocurrent. No significant effect of the reduction of light pulse duration on the linewidth and contrast of photocurrent resonances was observed (Supplementary Fig. 4a). The detection of the photocurrent induced by light pulses as short as 50 ns was in addition achieved using a charge sensitive preamplifier instead of the lock-in technique (Supplementary Fig. 4b and Supplementary Fig. 5a and b). This demonstrates the possibility to perform a fast readout of the photocurrent and shows that the photoelectric detection method may be used for pulsed spin-resonance experiments, for which readout laser pulses between 200 and 500 ns are typically used^43^,^44^.

In conclusion, we have demonstrated a new principle for the readout of NV^−^ centres spin magnetic resonances in diamond. By reducing the contact area, this technique has the potential to address single NV^−^ centres. This paradigm may lead to a sensitive way for the construction of diamond nanoscale sensors and quantum devices and their readout, allowing directly performing quantum operation on a chip.

## Methods

### Experimental setup

The samples characterized by ODMR and PDMR were prepared by electron-irradiating a high-pressure high-temperature type*-*Ib single-crystal diamond (2.9 × 2.9 × 0.5 mm^3^ plate from Element Six) with an initial concentration of approximately 200 p.p.m. of N_s_^0^ (sample E2) and a chemical vapour deposited optical grade type-IIa single-crystal diamond (2.8 × 2.8 × 0.28 mm^3^ plate from Element Six) with an initial concentration of N_S_^0^ below 1 p.p.m. (sample E7). Electron irradiation at 14 MeV with doses of 10^18^ and 10^16^ cm^−2^ for type-Ib and type-IIa samples, respectively, was performed at the Mainz Microtron. After electron irradiation, the samples were annealed for 4 h at 700 °C in vacuum, leading to a concentration of NV^−^ centres of ∼20 p.p.m. in E2 and ∼10 p.p.b. in E7. Before the fabrication of electrodes, the diamond crystals were cleaned and oxidized for 30 min in an acidic mixture of H_2_SO_4_ and KNO_3_ at 250 °C, and rinsed with ultrapure water. Coplanar interdigitated electrodes with a distance of 15 or 100 μm were then prepared on one of the faces of the diamond crystals by lift-off photolithography and sputtering of 20 nm of titanium topped by 80 nm of aluminium. After metal sputtering, the samples were annealed in vacuum at 510 °C for 3 h to form Ohmic contact between diamond and electrodes.

For ODMR and PDMR measurements, the 532 nm illumination is produced by a linearly polarized single-mode Nd:YAG Laser (Gem 532 from Quantum Laser).

For measurements on sample E2, this light is pulsed by an acousto-optical modulator (AOM; model 3200-146 from Crystal Technology) with a diffraction efficiency of 80 % and a contrast ratio of 1,000:1. Except for the study of the influence of light pulse duration on ODMR and PDMR signal, all the measurements were performed with a pulsing frequency of 531 Hz. The pulsed light beam is focused onto the diamond surface using a × 10 air objective with a numerical aperture of 0.28 and a working distance of 33.5 mm, resulting in a light spot with a diameter around 7 μm. This 7 μm light spot is positioned in the 15 or 100 μm gap separating the coplanar electrodes. Photoluminescence is measured in a non-confocal configuration. Photoluminescence light is collected by the same objective and filtered using a dichroic mirror with a cut-on wavelength of 552 nm and a sharp-edge long-pass filter with a cut-on wavelength of 550 mm (optical density >5 in the rejection region). The photoluminescence light is focused onto a pyroelectric detector. The output voltage of the pyroelectric detector is measured with a lock-in amplifier (7260 DSP from EG&G), referenced to the AOM pulsing (time constant: 50 ms).

For measurements on sample E7, the 532 nm light is pulsed at a frequency of 531 Hz using a chopper wheel. The pulsed light beam is focused onto the diamond surface using an air objective with a numerical aperture of 0.82 and a working distance of 400 μm, resulting in a light spot with a diameter ∼0.8 μm. This light spot is positioned in the 15 μm gap separating the coplanar electrodes. The photoluminescence light is measured by an avalanche photodiode.

For photocurrent measurements on samples E2 and E7, the photo-carriers generated upon green illumination are driven towards electrodes by applying a DC voltage between coplanar contacts. For light pulses between 10 and 940 μs, the photocurrent is amplified by a low-noise current to voltage preamplifier (SR570 from SRS, gain: 20 nA V^−1^), and measured using a lock-in amplifier (SR830 from SRS) referenced to the AOM or chopper wheel frequency (time constant: 30 ms). The settings of the lock-in amplifier are chosen so that the magnitude of the signal vector (which does not depend on the phase between the signal and lock-in reference) is recorded. We measure therefore the photocurrent intensity, independent of the sign of the photocurrent. Lock-in amplification leads to high signal-to-noise ratio. The photocurrent measured in our experiments was in the range of 10 pA to 1 nA and was detected with a four-digit precision. For fast photocurrent measurements (light pulses between 50 and 400 ns), the photocurrent is amplified using a charge sensitive preamplifier with a rise time of 2.5 ns and a sensitivity of 4 V/pC (A250CF CoolFET from AMPTEK INC.). The high sensitivity of this amplifier allows the detection of 48 electrons in a single pulse. The output signal of the preamplifier is measured using a digital real time oscilloscope (TDS 620B from Tektronix, bandwidth: 500 MHz). The DC offset of the charge sensitive preamplifier was subtracted from the measured signal to obtain the data presented in Supplementary Figs 4b and 5a,b. The presented signals were in addition smoothed to remove high-frequency noise.

Microwaves of controlled frequency are produced with a 200–4,000 MHz radio-frequency signal generator (TEG4000-1 from Telemakus) with an output power of 1 mW. The microwave power is set to 40 mW (Fig. 5a), 1W (Supplementary Figs 2 and 3a,b) or 5 W (Figs 1b and 4, Supplementary Figs 1, 4a and 6a,b) using an attenuator (TEA4000-3 from Telemakus) and a broadband amplifier with a gain of 45 dB (ZHL-16W-43+ from Mini-circuits). The microwave field is applied using a metal wire pressed across the diamond surface and connected to a 50 Ω terminator. The metal wire is insulated from coplanar electrodes by a polymer coating.

The static magnetic field is applied using a permanent neodymium magnet. Based on the relation between the splitting between ODMR resonances and the magnetic field magnitude^8^, the magnitude of the magnetic field applied along the [100] is estimated to be 0.50 mT and the amplitude of the magnetic field applied along the [111] direction to be 2.0 mT.

For ODMR and PDMR measurements, the microwave frequency is swept in the range 2,800–2,940 MHz with a step of 1 MHz. For each measurement, 100–200 spectra are recorded and averaged.

### Fitting of ODMR and PDMR spectra

To determine the splitting between magnetic resonances at zero-magnetic field (zero-field splitting, or ZFS), the FWHM and the contrast, PDMR and ODMR spectra recorded in the absence of external magnetic field were fitted to the sum of two Lorentzian functions:


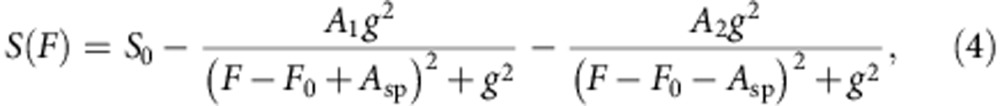


where *F* is the microwave frequency, *S*_0_ is the photocurrent or the photoluminescence intensity outside of resonances, *A*_1_ and *A*_2_ the amplitudes of respectively the first and the second magnetic resonances, *F*_0_ the central frequency, *A*_sp_ the ZFS and 2*g* the FWHM. The contrast presented in Fig. 4 corresponds to the amplitude *A*_2_ of the second zero-magnetic field resonance (situated at *F*_2_ = *F*_0_ + *A*_sp_ ∼2,873 MHz). Examples of ODMR and PDMR spectra fitting are presented in the Supplementary Fig. 6a,b and the corresponding fitting parameters are presented in the Supplementary Table 1.

### Evaluation of the electric field

Although the voltage applied in between coplanar electrodes results in a partially non-uniform electric field, a simple estimation of the magnitude of the applied electric field *F*_app_ is obtained by dividing the applied voltage *U* by the distance *L* in between electrodes. The magnitude of the local electric field in diamond is calculated using the Lorentz local field approximation^45^. In this approximation, the local electric field *F*_loc_ can be expressed as


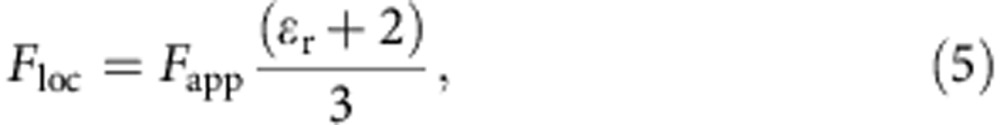


where *ɛ*_r_ is the relative electric permittivity of diamond.

## Additional information

**How to cite this article:** Bourgeois, E. *et al.* Photoelectric detection of electron spin resonance of nitrogen-vacancy centres in diamond. *Nat. Commun.* 6:8577 doi: 10.1038/ncomms9577 (2015).

## Supplementary Material

Supplementary Information Supplementary Figures 1-6, Supplementary Table 1, Supplementary Notes 1-4 and Supplementary References
