# Supplementary material for: Photoelectric detection of electron spin resonance of nitrogen-vacancy centres in diamond
Source: Nat Commun. 2015 Oct 21;6:8577. doi: 10.1038/ncomms9577 (PMC4639813; doi:10.1038/ncomms9577)
Supplement: Supplementary Information — Supplementary Figures 1-6, Supplementary Table 1, Supplementary Notes 1-4 and Supplementary References [file ncomms9577-s1.pdf]

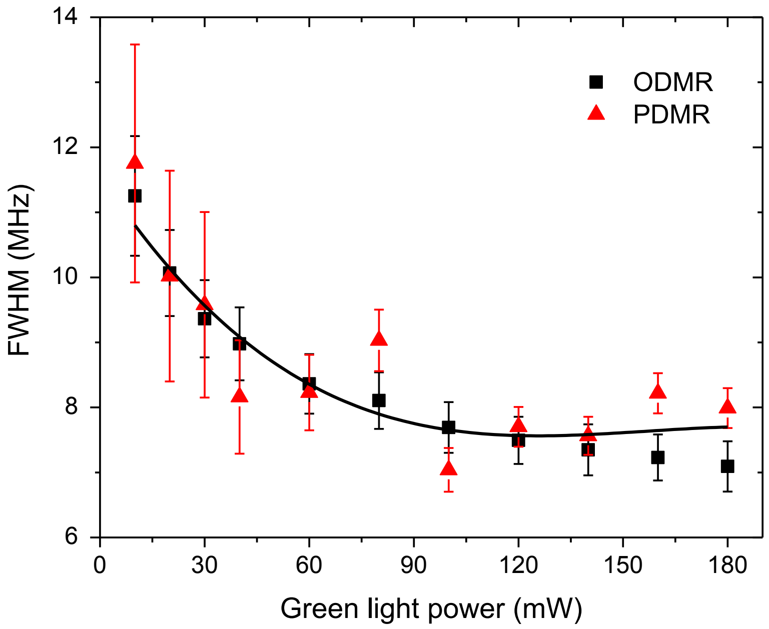

Supplementary Figure 1: Light power dependence of PDMR and ODMR full widths at half maximum (FWHM). Sample E2, applied electric field: 5 10^4 V cm^{-1}, light pulse duration: 940 µs, distance between contacts: 100 µm. Error bars represent the standard errors of the fitted values (see Methods for details on the fitting procedure). The solid line is a guide for the eye.

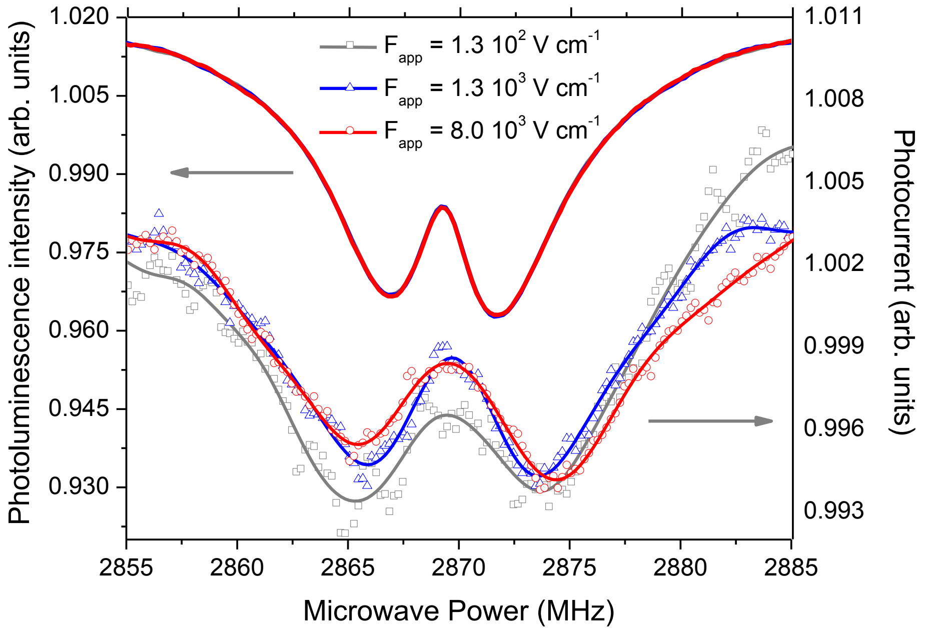

Supplementary Figure 2: Influence of the externally applied electric field on ODMR and PDMR spectra. F_{app}: applied electric field. Sample E2, green light power: 100 mW, light pulse duration: 940 µs, distance between contacts: 15 µm.

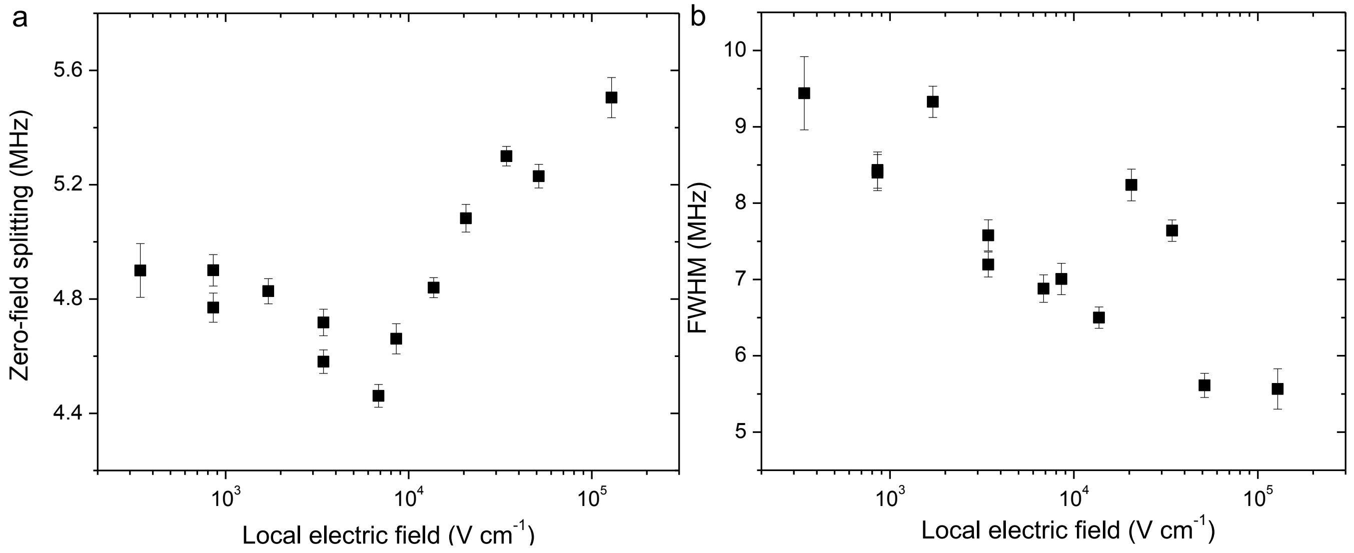

Supplementary Figure 3: Influence of the externally applied electric field on PDMR. (**a**) PDMR splitting at zero magnetic field, referred to as zero-field splitting (ZFS) and (**b**) PDMR full width at half maximum (FWHM) as a function of the magnitude of the local electric field. Sample E2, green light power: 100 mW, light pulse duration: 940 µs, distance between contacts: 15 µm. Error bars represent the standard errors of the fitted values (see Methods for details on the fitting procedure).

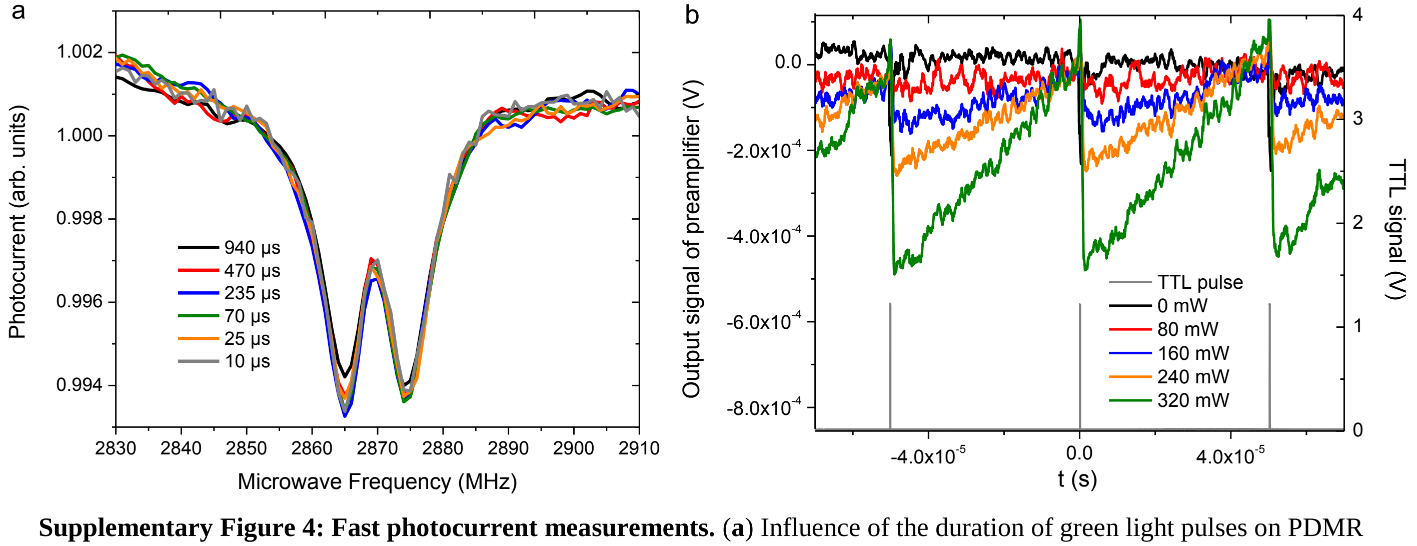

Supplementary Figure 4: Fast photocurrent measurements. (**a**) Influence of the duration of green light pulses on PDMR spectra (photocurrent amplified using a current to voltage preamplifier and measured by lock-in amplification). Sample E2, green light power: 100 mW, applied electric field: 5 10^4 V cm^{-1}, distance between contacts: 100 µm. (**b**) Time dependence of the photocurrent induced by illumination of diamond by 200 ns green light pulses at different green light powers. Sample E2, applied electric field: 5 10^4 V cm^{-1}, distance between contacts: 15 µm. The photocurrent is amplified using a charge sensitive preamplifier.

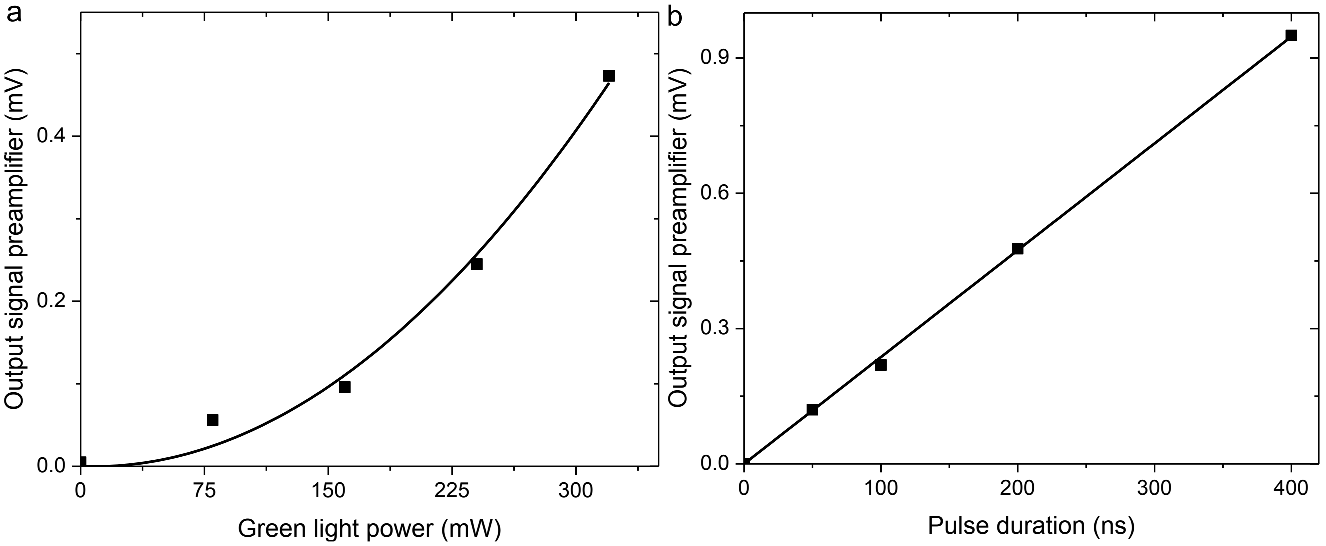

Supplementary Figure 5: Photocurrent induced by ultra-short light pulses. The absolute value of the minimum reached by the output voltage of the charge sensitive preamplifier, proportional to the photocurrent, is represented as a function of (**a**) the green light power (pulse duration: 200 ns) and (**b**) the light pulse duration (green light power: 400 mW). Sample E2, applied electric field: 5 10^4 V cm^{-1}, distance between contacts: 15 µm.

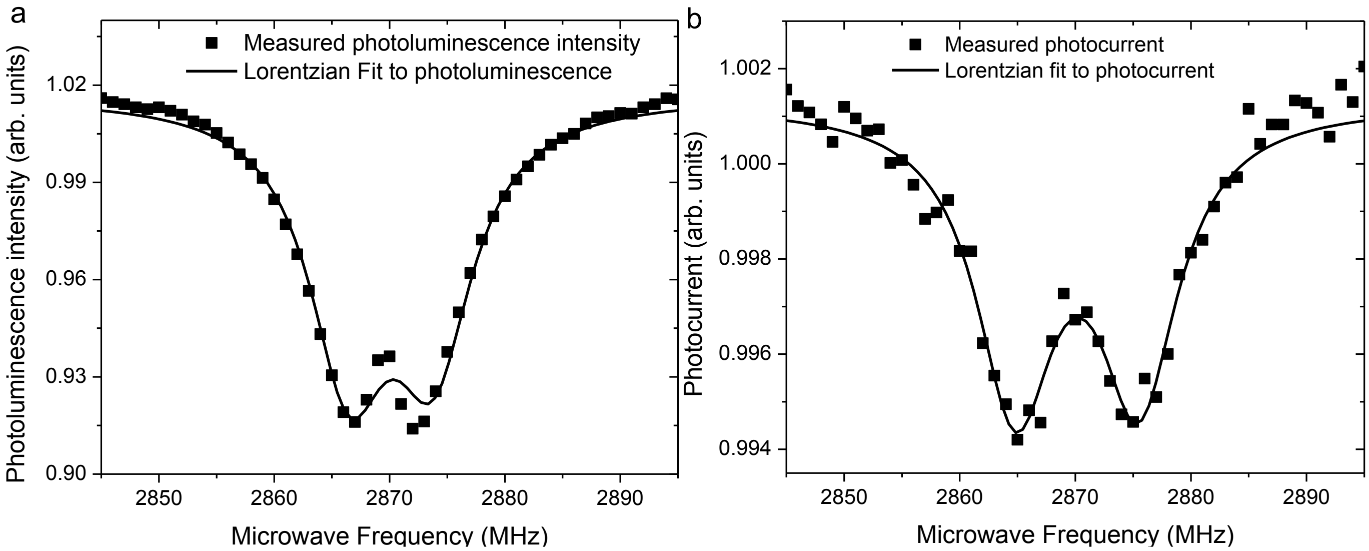

Supplementary Figure 6: Examples of fitted spectra. Experimental data and fitting of (**a**) ODMR and (**b**) PDMR spectra by the sum of two Lorentzian functions. Sample E2, green light power: 60 mW, applied electric field: 5 10^4 V cm^{-1}, light pulse duration: 940 µs, distance between contacts: 100 µm.

	Fitting parameters (ODMR)	Fitting parameters (PDMR)
**S_0 (arb. units)**	1.017(2)	1.0013(3)
**A_1 (arb. units)**	8.1(2) 10^{-2}	6.2(5) 10^{-3}
**A_2 (arb. units)**	7.4(1) 10^{-2}	5.4(4) 10^{-3}
**F_0 (MHz)**	2870.1(3)	2870.1(7)
**A_{sp} (MHz)**	3.71(8)	5.3(1)
**g (MHz)**	4.2(2)	4.1(3)
**R_0**	0.98617	0.95498

Supplementary Table 1: Example of fitting parameters obtained by fitting normalized ODMR and PDMR spectra. Experimental data are fitted by the sum of two Lorentzian functions. Sample E2, green light power: 60 mW, applied electric field: 5 10^4 V cm^{-1}, light pulse duration: 940 µs, distance between contacts: 100 µm. R_0 is the adjusted coefficient of determination.

Supplementary Note 1. Influence of strain and electric field on PDMR (type-Ib diamond).

The Supplementary Figure 2 depicts the influence of the applied electric field on normalized ODMR and PDMR spectra measured on sample E2.

No significant influence of the electric field magnitude on the position of the central resonance frequency F_0 (reflecting the splitting between the |0> and the |±1> spin sublevels) has been observed.

The influence of the magnitude of the local electric field on the splitting between magnetic resonances at zero-magnetic field (hereafter referred to as zero-field splitting or ZFS), determined by fitting PDMR spectra measured under different electric fields, is presented in Supplementary Fig. 3a. While the ZFS extracted from ODMR spectra (~ 3.0 MHz) is not affected by the electric field (Supplementary Fig. 2), the ZFS of PDMR varies between 4.4 and 5.5 MHz depending on the electric field magnitude.

The higher ZFS value and the electric-field dependence of the PDMR ZFS seem to indicate that photoluminescence (ODMR) and photocurrent (PDMR) measurements address different volumes of diamond material, with different strain. In OMDR, since measurements on sample E2 were performed using a non-confocal set-up, photons are emitted from the entire illumination cone, in which a low strain can be expected in average, and on which the influence of the externally applied electric field is negligible. On the contrary, PDMR addresses mostly an ensemble of NV^- centers located close to the diamond surface. Considering a charge carrier mobility[1] of $3.6\ 10^2$ cm^2 V^{-1} s^{-1} and a lifetime[2] below 3 ns, the maximal depth of charge collection varies from a few tens of nm below the surface for an applied electric field of $1.3\ 10^2$ V cm^{-1} to ~ 8 to 20 µm below the surface for an applied electric field of $5\ 10^4$ V cm^{-1}. Charge carriers created deeper in the sample are not collected to electrodes and therefore do not contribute to the measured photocurrent. The higher strain at the diamond surface than in the bulk of diamond most probably explains the higher ZFS detected in PDMR than in ODMR. In addition, for an identical applied electric field ($5\ 10^4$ V cm^{-1}), we observed a higher ZFS for PDMR spectra measured with an inter-electrode distance of 15 µm [ZFS = 5.51(7) MHz] than for PDMR spectra measured with an inter-electrode distance of 100 µm [ZFS = 5.23(7) MHz]. Since at high electric field the maximal depth of collection corresponds to the depth of penetration of the electric field in diamond, which increases with the inter-electrode distance, this result is in good agreement with the hypothesis of a higher strain close to the diamond surface.

It can be observed on Supplementary Fig. 3a that the ZFS of PDMR spectra presents a minimum for a local electric field of approximately $8\ 10^3$ V cm^{-1}. This electric-field dependence suggests the existence of two phenomena having opposing effects on the zero-field splitting.

On one hand, increasing the applied electric field results in an increase in the maximal depth of charge carrier collection (as long as the maximal depth allowing the collection of charge carriers at electrodes before their recombination remains shorter than the penetration depth of the electric field in diamond). An increased proportion of NV^- centers situated in the bulk of diamond – and thus experiencing lower strain – contributes therefore to the photocurrent signal. This might explain the decrease in the ZFS observed for local electric fields between $3.4\ 10^2$ and $8\ 10^3$ V cm^{-1}.

On the other hand, as mentioned in the main text, an increase in the magnitude of the electric field perturbs the |±1> spin manifold due to the Stark effect. To assess what would be the ZFS at low electric field if the charge collection volume was not affected by the electric field, we extrapolated the increase in ZFS observed above $8\ 10^3$ V cm^{-1} to low electric fields. This extrapolation leads to a ZFS of ~ 3 MHz at $3.4\ 10^2$ V cm^{-1}, similar to the ZFS observed on ODMR spectra. Based on this extrapolated value, we estimate an increase in the measured ZFS of ~ 2.5 MHz between $3.4\ 10^2$ and $1.3\ 10^5$ V cm^{-1}. To evaluate the influence

of the Stark effect on the ZFS in the applied range of electric field, we consider in first approximation the electric field as aligned with the axis of NV^- centers, although the 100-oriented sample under study contains NV^- centers of different orientations, forming various angles with the applied electric field. Considering a transverse electric susceptibility parameter[3] of 17 Hz cm V^{-1}, an increase of the ZFS of 2.2 MHz is expected when the local field varies from 3.4 10^2 to 1.3 10^5 V cm^{-1}. This seems to indicate that the Stark effect could explain the increase in ZFS observed for local electric fields above 8 10^3 V cm^{-1}.

The electric-field dependence of the PDMR linewidth is depicted in Supplementary Fig. 3b. While the FWHM of ODMR does not vary significantly with the electric field (Supplementary Fig. 2), the linewidth of PDMR spectra decreases by a factor 1.7 between 1.3 10^2 V cm^{-1} and 5 10^4 V cm^{-1}. At high electric field the PDMR linewidth (5.5 MHz) becomes very close to the ODMR linewidth (5 MHz) at identical microwave power (1 W at the output of the microwave amplifier). In diamond containing ensembles of NV^- centers, the total linewidth of optically detected electron spin resonances is the sum of the intrinsic broadening, determined by the spin properties of the sample (longitudinal and transverse spin relaxation times, Rabi frequency), and the inhomogeneous broadening, due to variations in the local environment of the different NV centers in the ensemble[4]. A first explanation of the electric-field induced narrowing of PDMR resonances might be the reduction of the inhomogeneous broadening. Indeed, as mentioned in the previous paragraph, due to a shorter charge carrier collection depth, PDMR measurements performed at low electric field address a higher proportion of NV^- centers located closer to the diamond surface. At proximity of the surface, the local environment is more likely to vary between different NV centers than deeper in the bulk, leading to a higher inhomogeneous broadening. The broader lines observed at low electric field could also result from a stronger dispersion in the charge carriers transport times, due to trapping of charge carriers by impurities in the crystal and subsequent detrapping.

Supplementary Note 2. Comparison beween ODMR and PDMR linewidths (type-Ib diamond).

The full widths at half maximum (FWHM) of PDMR spectra recorded on sample E2 at different green light powers in the absence of external magnetic field are presented in the Supplementary Fig. 1. FWHM between 7.5 and 11.5 MHz are observed for ODMR spectra, close to the linewidths previously reported for ensembles of NV centers in electron-irradiated type-Ib diamond[4,5]. PDMR spectra present identical FWHM. This demonstrates that in the case of measurements performed under high applied electric field (5 10^4 V cm^{-1}) the photoelectric detection of spin magnetic resonances does not lead to any broadening of the resonances. A decrease of 35 % in the linewidths of both ODMR and PDMR is observed when the green light power is increased from 10 to 180 mW. This result is in agreement with the light narrowing of microwave power-broadened magnetic resonances described in [[4]] and attributed to a decrease in the ratio between the longitudinal and the transverse spin relaxation times at high light power[4].

Supplementary Note 3. PDMR sensitivity.

The derivation of the Equation (1) of the main text is detailed in this Supplementary Note. In a first approximation, the magnitude of the electric field is considered as uniform in the diamond crystal and the field lines are considered as half ellipses extending between electrodes. The photocurrent associated with the ionization of NV^- centers (quadratic fraction of the photocurrent) i_{NV} can be expressed as:

$$i_{\mathrm{NV}} = e\,\gamma(G - R), \text{ Supplementary Equation (1)}$$

where e is the elementary charge (C), γ the photoelectric gain, G the rate of charge generation (s^{-1}) and R the rate of charge recombination (s^{-1}).

Based on the expression of the two-photon ionization rate established in[6], the generation rate G can be expressed as:

$$G = \frac{\eta N_{\mathrm{p}}^2 n_{\mathrm{NV}} V_1}{\Gamma}, \text{ Supplementary Equation (2)}$$

where η is the efficiency of charge carrier creation, N_{p} is the number of incident photons per second (s^{-1}), n_{NV} the density of NV^- centers in the sample (cm^{-3}), V_1 the volume containing the NV^- centers contributing to the photocurrent (cm^3) and Γ the decay rate from NV^- excited state to NV^- ground state ($7 \cdot 10^7$ s^{-1}, considering a lifetime of 13.7 ns for the |0> spin sublevel of NV^- excited state[7]). The number of incident photons per second has been calculated from the light power, taking into account the fraction of the incident light reflected by diamond (17 % at 532 nm). The volume V_1 is delimited by the illumination cone and by the maximal depth of charge carrier collection *d*. This distance can be defined as the shortest value between the depth of penetration of the electric field in the diamond material (~ 20 µm for coplanar electrodes separated by 15 µm[8]) and the maximal depth allowing the collection of charge carriers at electrodes before their recombination (which depends on the mean free path of charge carriers). Taking into account this volume and the average density of NV^- centers, we estimate that ~ $3 \cdot 10^9$ NV^- centers contribute to the photocurrent in sample E2 and ~ $3 \cdot 10^6$ in sample E7.

In high-mobility materials, such as diamond, the recombination of charge carriers is dominated by trap-assisted recombination, described by the Shockley-Read-Hall model[9,10]. In this case, the rate of recombination R can be expressed as a function of the density of charge carriers n (cm^{-3}), the lifetime of charge carriers τ (s) and the volume V_2 in which the drift of free charge carriers takes place (cm^3)[9]:

$$R = \frac{n V_2}{\tau}. \text{ Supplementary Equation (3)}$$

A high estimation of the recombination rate is obtained by deriving the charge carrier density from the density of current flowing out of the charge generation volume V_1, and by considering the density of charge carriers as constant in the volume V_2. The density of charge carriers can then be expressed as:

$$n = \frac{2G}{\mu S F_{\mathrm{app}}}, \text{ Supplementary Equation (4)}$$

where μ is the charge carrier mobility ($cm^2 \cdot V^{-1} \cdot s^{-1}$), F_{app} is the applied electric field ($V \cdot cm^{-1}$) and S the axial cross section (cm^2) of the generation volume V_1.

Using Supplementary Equations (1) to (4), the fraction i_{NV} of the measured photocurrent associated with the ionization of NV^- centers can finally be expressed as:

$$i_{\mathrm{NV}} = \frac{e \gamma \eta N_{\mathrm{p}}^2 n_{\mathrm{NV}} V_1}{\Gamma} \left(1 - \frac{2 V_2}{\tau \mu S F_{\mathrm{app}}}\right). \text{ Supplementary Equation (5)}$$

Supplementary Note 4. Fast photocurrent measurements (type-Ib diamond).

To demonstrate the possibility to use the photoelectric detection technique to perform pulsed spin detection experiments (measurement of Ramsey or Rabi oscillations, spin-echo experiments, etc.), the photocurrent induced by light pulses shorter than 1 ms was measured.

PDMR spectra obtained for different light pulse durations are presented on Supplementary Fig. 4a. For these measurements, the photocurrent is amplified using a current to voltage preamplifier and measured by lock-in amplification. A drop in the photocurrent by a factor 60 is observed when the pulse duration is reduced from 940 to 10 µs, due to the decrease in the effective gain of the current-to-voltage preamplifier at high frequency. However, it appears that reducing the duration of light pulses down to 10 µs does not affect significantly the contrast or the linewidth of photocurrent resonances (Supplementary Fig. 4a). This experiment demonstrates therefore the possibility to perform the photoelectric detection of spin magnetic resonances using µs light pulses.

Due to the limited bandwidth of the current-to-voltage preamplifier at high gain, pulsed photocurrent measurements with light pulses shorter than 10 µs could not be performed using the same setup. For faster measurements (light pulses between 50 and 400 ns), a charge sensitive preamplifier was used (see Methods for more details about the setup). The charge sensitive preamplifier provides an output voltage proportional to the input charge. Supposing a constant input current I, the time dependence of the output voltage V can therefore be expressed as:

$$V\,(\mathrm{t}) = -\alpha\, I\, t,$$ Supplementary Equation (6)

where α is the sensitivity of the preamplifier.

The Supplementary Fig. 4b depicts the time evolution of the output voltage of the preamplifier under illumination of diamond by 200 ns green light pulses of different powers. Two consecutive light pulses are separated by 50 µs. The photocurrent pulses are converted to voltage pulses by the charge sensitive preamplifier. After application of a light pulse, the collection of photo-carriers produced in the sample induces a fast decrease in the output voltage of the preamplifier. Although the charge preamplifier we used (see Methods) has a short rise time (2.5 ns), it presents an intrinsic decay time of 500 µs. After reaching a minimum, the output voltage increases therefore slowly, and decreases sharply again when the following light pulse illuminates the sample.

According to formula (6), for a fixed light pulse duration, the minimum voltage reached at the output of the preamplifier is proportional to the photocurrent induced in the sample by the light pulse. The absolute value of the minimum reached by this output voltage for different laser powers is plotted in the Supplementary Fig. 5a. As expected, we observe an increase in the absolute value of this minimum voltage as the power of the laser increases. The minimum reached by the output voltage after each light pulse appears in addition to increase linearly with the light pulse duration (Supplementary Fig. 5b), which is in good agreement with formula (6). Light pulses as short as 50 ns induce a decay in the output voltage of 0.12 mV, still clearly detectable on the oscilloscope trace.

These results prove the possibility to detect the charge induced by light pulses with duration in the range of tens to hundreds of ns. This confirms that the photoelectric method may be used to perform pulsed spin experiments, for which read-out laser pulses between 200 to 500 ns are typically used[11,12].

Supplementary References:

1. Malinauskas T. *et al.*, Optical evaluation of carrier lifetime and diffusion length in synthetic diamonds. *Diamond Relat. Mater.* **17**, 1212-1215 (2008)
2. Scajev P., Gudelis V., Ivakin E., Jarasiunas K., Nonequilibrium carrier dynamics in bulk HPHT diamond at two-photon carrier generation. *Phys. Status Solidi A* **208**, 2067-2072 (2011)
3. Van Oort E. and Glasbeek M., Electric-field-induced modulation of spin echoes of N-V centers in diamond. *Chem. Phys. Lett.* **168**, 529-532 (1990)
4. Jensen K., Acosta V. M., Jarmola A., Budker D., Light narrowing of magnetic resonances in ensembles of nitrogen-vacancy centers in diamond. *Phys. Rev. B* **87**, 014115 (2013)
5. Kim E., Acosta V. M., Bauch E., Budker D., Hemmer P. R., Electron spin resonance shift and linewidth broadening of nitrogen-vacancy centers in diamond as a function of electron irradiation dose. *Appl. Phys. Lett.* **101**, 082410 (2012)
6. Siyushev P. *et al.*, Optically controlled switching of the charge state of a single nitrogen-vacancy center in diamond at cryogenic temperatures. *Phys. Rev. Lett.* **110**, 167402 (2013)
7. Robledo L., Bernien H., van der Sar T., Hanson R., Spin dynamics in the optical cycle of single nitrogen-vacancy centres in diamond. *New J. Phys.* **13**, 025013 (2011)
8. Dong T. and Barbosa C., Capacitance variation induced by microfluidic two-phase flow across insulated interdigitated electrodes in lab-on-chip devices. *Sensors* **15**, 2694-2708 (2015)
9. Nelson J., *The physics of solar cells* [Imperial College Press] (London, 2003)
10. Mizuochi N. *et al.*, Electrically driven single-photon source at room temperature in diamond. *Nature Photonics* **6**, doi:10.1038 (2012)
11. Laraoui A. and Meriles C.A., Rotating frame spin dynamics of a Nitrogen-Vacancy center is a diamond nanocrystal. *Phys. Rev. B* **84**, 161403 (2011)
12. Stepanov V., Cho F. H., Abeywardana C., Takahashi S., High-frequency and high-field optically detected magnetic resonance of nitrogen-vacancy centers in diamond. *Appl. Phys. Lett.* **106**, 063111 (2015)
